# Acute β-cell failure induced by selpercatinib in RET fusion–positive non-small cell lung cancer: A case report

**DOI:** 10.1016/j.rmcr.2026.102372

**Published:** 2026-01-19

**Authors:** Hitokazu Tsukao, Ryosuke Kojima, Shinichi Nakanishi, Yudai Miyanishi, Yuya Fujii, Wataru Yamaguchi, Junya Nakaya, Toru Kojima

**Affiliations:** Department of Respiratory Medicine, Fukui Prefectural Hospital, 2-8-1 Yotsui, Fukui City, Fukui, 9108526, Japan

## 1 Introduction

Lung cancer remains the leading cause of cancer-related mortality worldwide, and over 70,000 people die from the disease each year in Japan [1]. Non-small cell lung cancer (NSCLC) accounts for approximately 85 % of all cases of lung cancer, with adenocarcinoma being the most common histological type [2]. Recent advances in comprehensive genomic profiling have enabled the identification of actionable oncogenic drivers, allowing the use of molecularly targeted agents capable of significantly improving long-term outcomes.

The RET (*rearranged during transfection*) protooncogene is aberrantly activated by point mutations or gene fusions in approximately 2 % of all malignancies [3]. Although activating RET mutations are most frequently observed in medullary thyroid carcinoma (MTC), RET fusions occur in 5 %–10 % of papillary thyroid carcinomas (PTC) and 1 %–2 % of NSCLC cases [4,5].

Supported by the pivotal LIBRETTO-001/431 trials [6,7], selpercatinib received FDA accelerated approval for RET fusion NSCLC in the United States in 2020 and national reimbursement in Japan in 2021. Metabolic toxicity has rarely been reported. In LIBRETTO-001, hyperglycemia of any grade occurred in 5.3 % of treated patients, with grade 3–4 events in 2.8 % of cases; however, no such events were reported in LIBRETTO-431. Conversely, the LIBRETTO-531 trial, which investigated 193 patients with RET-mutant MTC, documented one case of diabetic ketoacidosis requiring treatment discontinuation [8].

Selpercatinib's Japanese package insert does not mention hyperglycaemia, and no cases have been reported in domestic NSCLC studies. To the best of our knowledge, there is no published Japanese case report describing selpercatinib-associated hyperglycemia in NSCLC. Herein, we present a patient with preexisting type 2 diabetes who developed rapidly progressive insulin secretory failure shortly after initiating selpercatinib therapy.

## 2 Case presentation

A 66-year-old man with type 2 diabetes mellitus (on metformin plus a low-dose DPP-4 inhibitor) was referred for the management of a productive cough that persisted for six months. His initial chest CT revealed a right lower lobe mass, and staging scans confirmed stage IVB lung adenocarcinoma with pulmonary, pleural, osseous, and bilateral adrenal metastases. Targeted next-generation sequencing using the Oncomine™ Precision Assay (tumor type: non-small cell lung cancer) identified a KIF5B-RET fusion, and the programmed death-ligand 1 (PD-L1) tumor proportion score (22C3 antibody) was 20 %. The final diagnosis was advanced lung adenocarcinoma harboring a RET fusion.

**Fig. 1:**
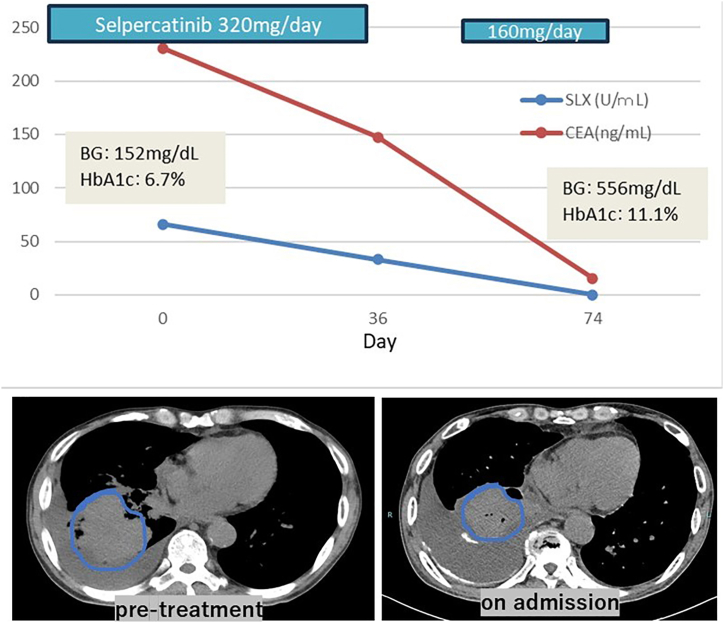
Trends in tumor markers (CEA and SLX), blood glucose, and HbA1c during selpercatinib treatment, along with representative axial CT images. The upper panel shows a timeline of tumor markers (CEA in ng/mL, SLX in U/mL) and glycemic parameters (blood glucose [BG] and HbA1c) across three time points: baseline (day 0), day 36, and day 74. Blue and red lines indicate SLX and CEA, respectively. Blood glucose and HbA1c levels at baseline and on admission are highlighted. The treatment course of selpercatinib is depicted, including the initial full dose (320 mg/day), temporary discontinuation, and subsequent reinitiation at a reduced dose (160 mg/day). The lower panel presents contrast-enhanced axial CT images before treatment and on admission. The circled areas indicate the target tumor site in the lower lobe of the left lung, which shows marked shrinkage, consistent with a partial response to therapy. Despite the tumor response, the patient developed acute hyperglycemia.

The baseline glycemic control was acceptable (random plasma glucose level: 152 mg/dL; HbA1c (NGSP): 6.7 %). Selpercatinib was initiated at a once daily dose of 320 mg. During the first treatment cycle, serum tumor marker levels declined, and follow-up imaging confirmed tumor shrinkage, indicating a favorable early response (Fig. 1). However, on day 30, the patient developed grade 3 hepatotoxicity, necessitating a 24-day drug interruption. After liver enzyme levels normalized, therapy was resumed at a two-step reduced dose of 160 mg/day.

Twenty days after the drug was reintroduced, liver levels remained within the normal range; however, during a routine outpatient visit, the patient complained of polydipsia and polyuria. Urinalysis revealed glycosuria (4+) and ketonuria (1+), and laboratory testing revealed a random plasma glucose level of 556 mg/dL and an HbA1c level of 11.1 %, indicating abrupt glycemic deterioration (Fig. 1). He had not received glucocorticoids during treatment, and there had been no recent changes in β-blocker or diuretic therapy, dietary intake, or physical activity. The results of various tests, including insulin secretion and insulin resistance, are shown in Table 1. Ultimately, the patient was diagnosed with an insulin secretion disorder. Intensive insulin therapy promptly stabilized his glycemia, after which selpercatinib was safely resumed at an intermediate dose of 240 mg/day without recurrence of hepatotoxicity or further metabolic complications. Taken together, the clinical course strongly suggests that selpercatinib precipitated the rapid worsening of glycemic control through the acute impairment of insulin secretion.

**Table 1:** Summary of glucose metabolism and insulin secretory function in the present case.

Test Item	Result
Plasma glucose (random)	556 mg/dL
HbA1c(NGSP)	11.10 %
Serum C-peptide	0.50 ng/mL
Serum insulin	4.3 μU/mL
ACTH	18.5 pg/mL
Cortisol	10.6 μg/dL
Anti-GAD antibody	<5.0 U/mL
Urinary C-peptide (24h)	14.6 μg/day
**Glucagon stimulation test**	
Serum C-peptide (CPR), baseline	0.43 ng/mL
Serum C-peptide (CPR), 6 min after glucagon	1.00 ng/mL
Plasma glucose, baseline	106 mg/dL
Plasma glucose, 6 min after glucagon	117 mg/dL

C-peptide (CPR) and insulin levels suggested decreased insulin secretory capacity. Although glucagon stimulation induced a modest increase in CPR, fasting glucose and CPR were not consistent with preserved β-cell function. The HOMA-IR value remained within normal limits, suggesting preserved insulin sensitivity and supporting a primarily secretory defect.

## 3 Discussion

Although RET fusion–positive lung cancer represents only 1 %–2 % of NSCLC cases, selpercatinib use is expanding across RET-altered tumors. Our case shows that, albeit rare, clinically significant hyperglycemia can occur during therapy. Malignancies may disrupt glucose homeostasis through tumor-associated insulin resistance and inflammation—chronic insulin/IGF1 activation can drive oncogenesis, while cytokines such as interleukin-6 and tumor necrosis factor-α blunt peripheral insulin sensitivity, predisposing to tumor-related hyperglycemia [9]. Likewise, cancer cachexia amplifies gluconeogenesis, lipolysis, and proteolysis, aggravating insulin resistance [10]. Beyond cachexia, cancer cells can worsen dysglycemia by reprogramming stromal and systemic metabolism through Warburg-driven paracrine signaling [11]. Such mechanisms usually parallel uncontrolled tumor growth; in our patient, however, the carcinoembryonic antigen titer decreased from 164.6 ng/mL to 15.6 ng/mL, and the sialylLewis X titer dropped from 66 U/mL to 37 U/mL, indicating effective disease control and making tumor-derived factors an unlikely etiology of hyperglycemia. The abrupt rise in the glucose level and the severe loss of insulin secretory capacity were therefore attributed to selpercatinib itself. In addition to its primary RET target, selpercatinib weakly inhibits FGFR 1/2/3 and VEGFR 1/3 at therapeutic concentrations [12]. FGFR signaling sustains β-cell survival and glucose-stimulated insulin secretion; fibroblast growth factors 1 and 21 enhance insulin release and regulate adipose metabolism via the ERK1/2 and Akt-dependent pathways [[13], [14], [15]]. Off-target FGFR blockade could thus trigger β-cell failure. Tyrosine kinase inhibitors that inhibit VEGFR have likewise been linked to hyperinsulinemia and insulin resistance, presumably via reduced endothelial nitric oxide production and the ensuing impairment of microvascular blood flow in skeletal muscle and adipose tissue [16]. Although both pathways may operate simultaneously, the blunted C-peptide response to glucagon in this case strongly suggests that β-cell dysfunction was the dominant mechanism.

To contextualize the rarity of selpercatinib-associated dysglycemia, we reviewed safety data from pivotal clinical trials and regulatory documents. As summarized in Table 2, laboratory “increased glucose” was frequently reported in the single-arm LIBRETTO-001 study, whereas it was not listed among common laboratory abnormalities in phase III trials (LIBRETTO-431 and LIBRETTO-531), likely reflecting differences in study populations and reporting thresholds. Importantly, diabetic ketoacidosis was reported as a fatal adverse event in the phase III LIBRETTO-531 trial, although no case-level clinical details were provided.

**Table 2:** Evidence of dysglycemia associated with selpercatinib from clinical trials and regulatory documents.

Source	Study/population	N	Event	Frequency/comment
FDA label [17]	LIBRETTO-001 (RET-altered tumors)	∼800	Increased glucose	Any grade 53 %; Grade ≥3 2.8 %
FDA label [17]	LIBRETTO-431 (RET fusion NSCLC)	261	Increased glucose	Not reported above table threshold
FDA label [17]	LIBRETTO-531 (RET-mutant MTC)	144	Increased glucose	Not reported above table threshold
WirthL. J et al., 2024 [8]	LIBRETTO-531	144	Diabetic ketoacidosis	Fatal adverse event: 1 case
Health Canada label [18]	Pooled safety	NR	Diabetic ketoacidosis	Led to permanent discontinuation

Data are derived from pivotal clinical trials and regulatory documents [8,17,18]. Reported frequencies vary across studies owing to differences in study populations, laboratory monitoring, and reporting thresholds. Absence of an event from a table does not indicate absence of occurrence.

To date, no published case report has described selpercatinib-associated dysglycemia with a detailed evaluation of insulin secretory function. In this context, the present case provides unique clinical insight into the potential mechanism of acute β-cell failure induced by selpercatinib.

Therefore, clinicians should regard hyperglycemia during selpercatinib therapy not merely as a manifestation of insulin resistance but also as a potential indicator of acute β-cell injury. Regardless of preexisting diabetes, serial monitoring of blood glucose and Cpeptide concentrations is advisable to identify early βcell failure and enable the prompt initiation of insulin therapy when required.

## CRediT authorship contribution statement

**Hitokazu Tsukao:** Writing – original draft, Investigation, Data curation, Conceptualization. **Ryosuke Kojima:** Resources, Investigation. **Shinichi Nakanishi:** Resources, Investigation. **Yudai Miyanishi:** Resources, Investigation. **Yuya Fujii:** Resources, Investigation. **Wataru Yamaguchi:** Writing – review & editing, Supervision. **Junya Nakaya:** Writing – review & editing, Supervision. **Toru Kojima:** Writing – review & editing, Supervision.

## Ethics statement

Written informed consent for the publication of this case and its accompanying images was obtained from the patient using the form provided by the Japanese Respiratory Society.

## Declaration of generative AI and AI-assisted technologies in the writing process

When preparing this manuscript, the author used ChatGPT (OpenAI) to enhance its clarity, grammar, and academic style. Thereafter, the author critically reviewed and edited it as required and takes full responsibility for the content of the manuscript.

## Funding

This work received no external funding.

## Declaration of competing interest

The authors declare that they have no known competing financial interests or personal relationships that could have appeared to influence the work reported in this paper.
